# Necrotizing Otitis Externa Due to Scedosporium sp.: A Case Report

**DOI:** 10.7759/cureus.112867

**Published:** 2026-07-17

**Authors:** Chaimaa Boujloud, Zakaria Essamhi, Adil Maleb, Azzedine Lachkar, Aziza Hami

**Affiliations:** 1 Laboratory of Parasitology and Mycology, Central Laboratory, Faculty of Medicine and Pharmacy, Centre Hospitalier Universitaire Mohammed VI, Oujda, MAR; 2 Department of Otolaryngology - Head and Neck Surgery, Faculty of Medicine and Pharmacy, Centre Hospitalier Universitaire Mohammed VI, Oujda, MAR; 3 Laboratory of Microbiology, Central Laboratory, Faculty of Medicine and Pharmacy, Centre Hospitalier Universitaire Mohammed VI, Oujda, MAR

**Keywords:** fungal infection, mycological diagnosis, necrotizing otitis externa, scedosporium sp, voriconazole

## Abstract

Necrotizing otitis externa (NOE) is a severe and potentially life-threatening infection of the external auditory canal, particularly affecting elderly diabetic or immunocompromised patients. *Pseudomonas aeruginosa* is the most frequently isolated pathogen. Among fungi, *Aspergillus* and *Candida* spp. are the most commonly implicated. Nevertheless, *Scedosporium* sp. has also emerged as a notable pathogen in NOE. We report the case of a 67-year-old woman with poorly controlled diabetes who presented with persistent right-sided otorrhea and otalgia, complicated by facial paralysis. Imaging revealed bone erosion consistent with NOE. Mycological analysis of ear swabs identified *Scedosporium* sp. This case highlights the importance of considering fungal pathogens in refractory cases of otitis externa, particularly in high-risk patients. It underscores the need for early mycological investigations to ensure appropriate antifungal therapy.

## Introduction

Necrotizing otitis externa (NOE) is an aggressive infection of the external auditory canal that can extend to the temporal bone and skull base. This condition most commonly affects elderly diabetic patients or immunocompromised individuals. It typically begins as a benign external otitis but may progress to osteomyelitis involving deep bony structures. The most common clinical manifestations include persistent otalgia, otorrhea, headache, and cranial nerve involvement. Pseudomonas aeruginosa is the most commonly isolated pathogen. However, fungal forms of NOE, which primarily affect immunocompromised hosts, are associated with a high risk of systemic dissemination and mortality [1]. The most commonly implicated fungi are *Aspergillus* and *Candida* spp.

Nevertheless, *Scedosporium* sp. has also emerged as a significant pathogen in NOE [2]. *Scedosporium* spp. are environmental filamentous fungi commonly found in human-influenced environments. Importantly, these species are known for their low susceptibility or intrinsic resistance to amphotericin B, making early identification of fungal pathogens essential for guiding appropriate antifungal therapy. In severe cases, *Scedosporium* infection may progress to skull base osteomyelitis. This progression has been linked to vascular complications, including mycotic aneurysms and cerebral infarctions, further complicating management [3]. This report aims to describe a rare case of NOE caused by *Scedosporium* sp. in a diabetic patient, highlight the diagnostic challenges posed by this uncommon fungal pathogen, and emphasize the importance of early mycological investigations and appropriate antifungal therapy in refractory cases.

## Case presentation

We report the case of a 67-year-old woman with a medical history of poorly controlled diabetes mellitus for the past 20 years, treated with insulin, and arterial hypertension managed with dual therapy (a calcium channel blocker and an ACE inhibitor). The illness began approximately three months before presentation, characterized by the gradual development of right-sided otalgia and otorrhea. The patient initially received local antibiotic therapy without significant clinical improvement. She was subsequently referred to our facility for specialized management.

Clinical examination revealed purulent right otorrhea, inflammatory stenosis of the right external auditory canal, and ipsilateral facial paralysis. An auricular swab was collected for bacteriological and mycological analysis.

Laboratory investigations revealed an inflammatory syndrome characterized by leukocytosis (10 150 leukocytes/μL) with neutrophil predominance and an elevated C-reactive protein (CRP) level of 117 mg/L. Glycated hemoglobin was also elevated at 8.1%, indicating poor glycemic control.

**Figure 1:**
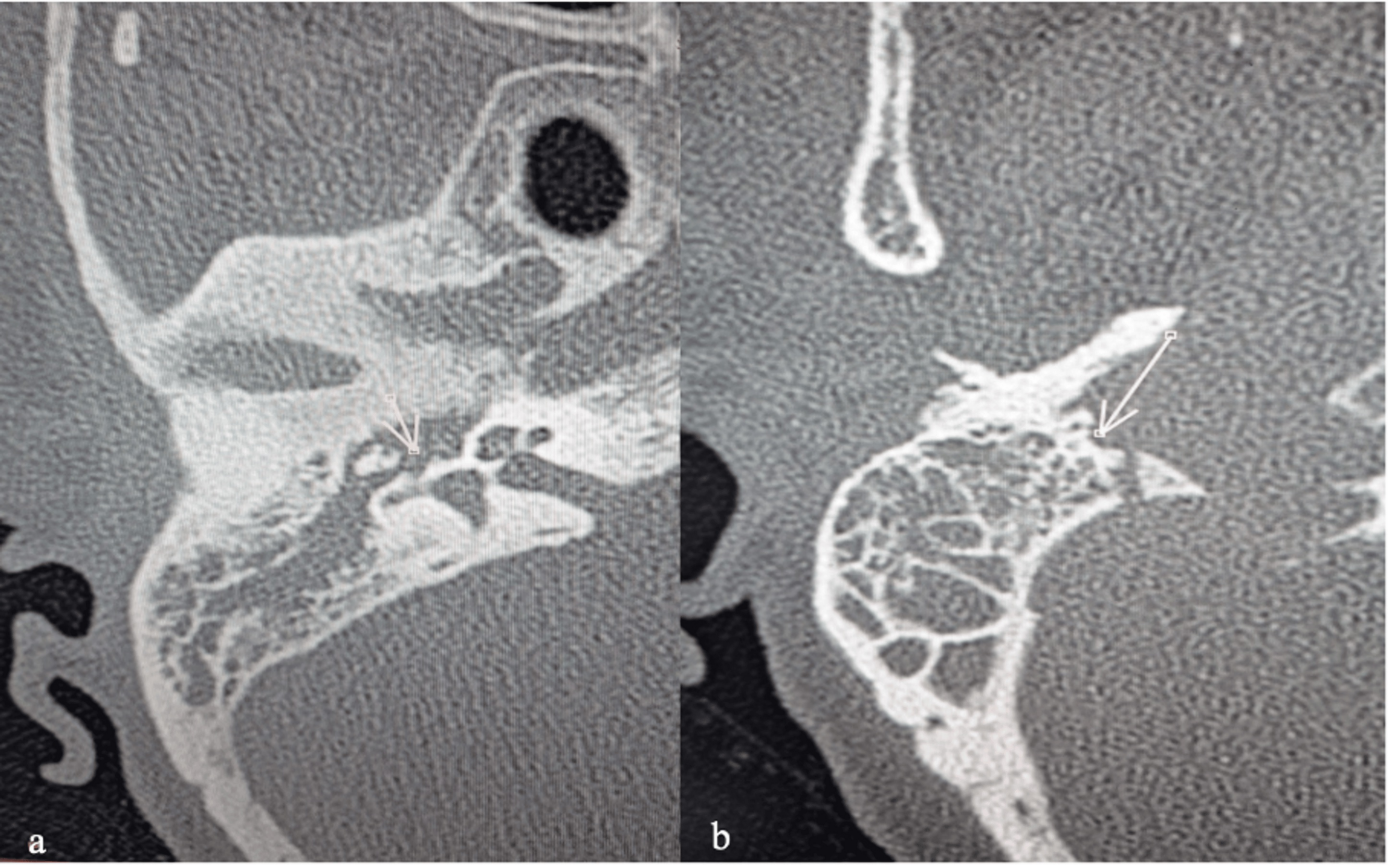
Skull base CT. (a) Axial image showing bone lysis of the anterior wall of the tympanic segment of the facial nerve canal. (b) Axial image showing bone lysis of the anterior wall of the mastoid CT: computed tomography

Skull base computed tomography (CT) supported the diagnosis of right-sided NOE, with evidence of lysis of the anterior wall of the tympanic segment of the facial nerve (Figure 1).

The patient was started on a combination therapy consisting of intravenous ceftriaxone 2 g every 24 hours, intravenous ciprofloxacin 400 mg every 12 hours, ciprofloxacin eye drops (five drops three times daily), injectable corticosteroids at 80 mg every 24 hours, and paracetamol 1 g every eight hours. Insulin therapy was initiated with a basal insulin regimen (8 IU at 9:00 p.m.) and rapid-acting insulin adjusted according to capillary blood glucose levels, with close monitoring of the glycemic profile. However, clinical re-evaluation showed no improvement in the patient’s condition, and CRP levels continued to rise, increasing from 117 to 187 mg/L, indicating progression of the inflammatory process.

**Figure 2:**
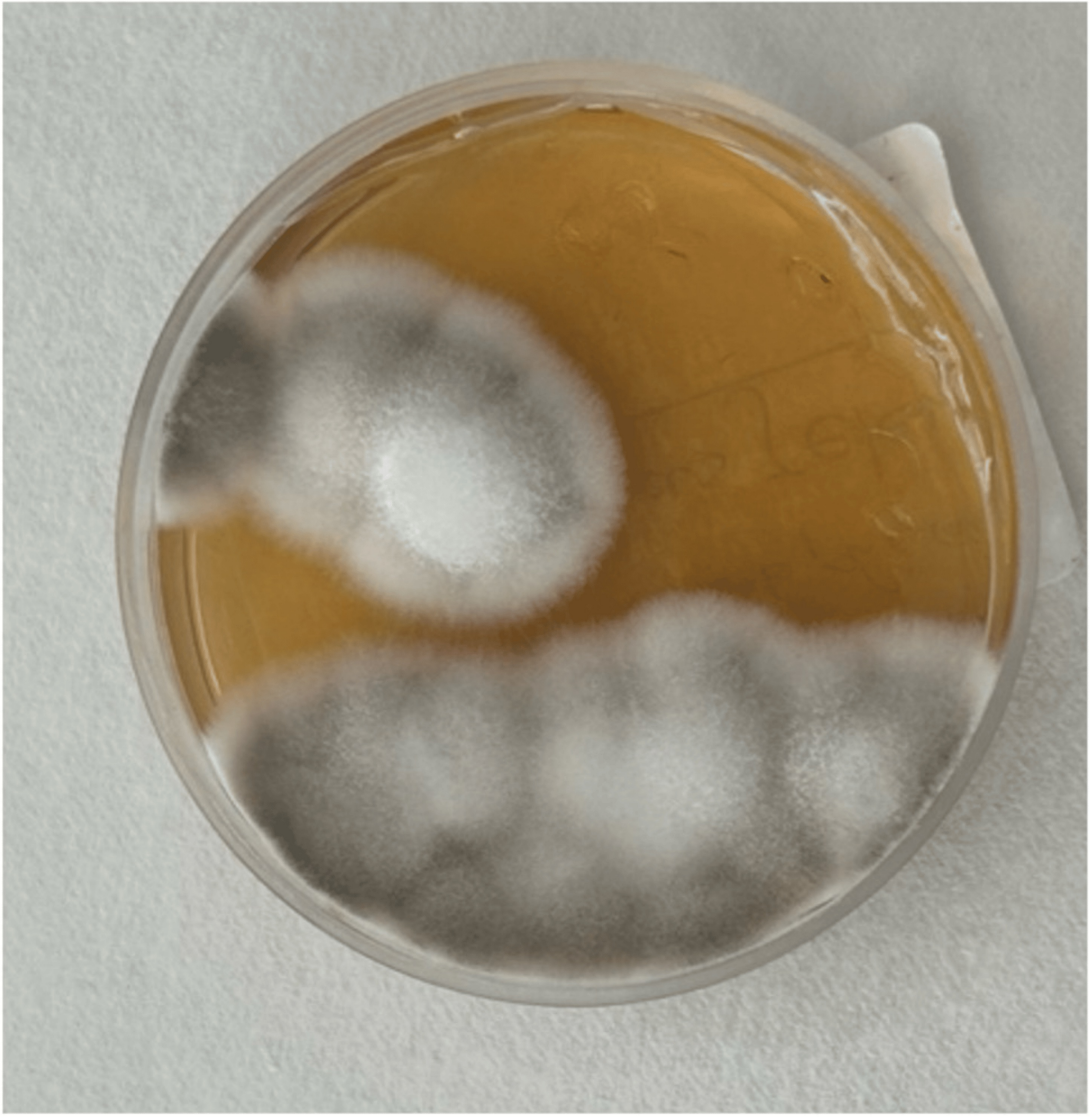
Macroscopic appearance of Scedosporium sp. colonies on SC agar after six days of incubation at 37°C SC: Sabouraud culture

The auricular swab was inoculated onto Sabouraud agar (SC) and Sabouraud chloramphenicol agar (SCA) culture media, which were then incubated at 25°C. Direct microscopic examination revealed the presence of fine, hyaline, septate hyphae. After two days of incubation, whitish to grayish cottony colonies were observed (Figure 2).

**Figure 3:**
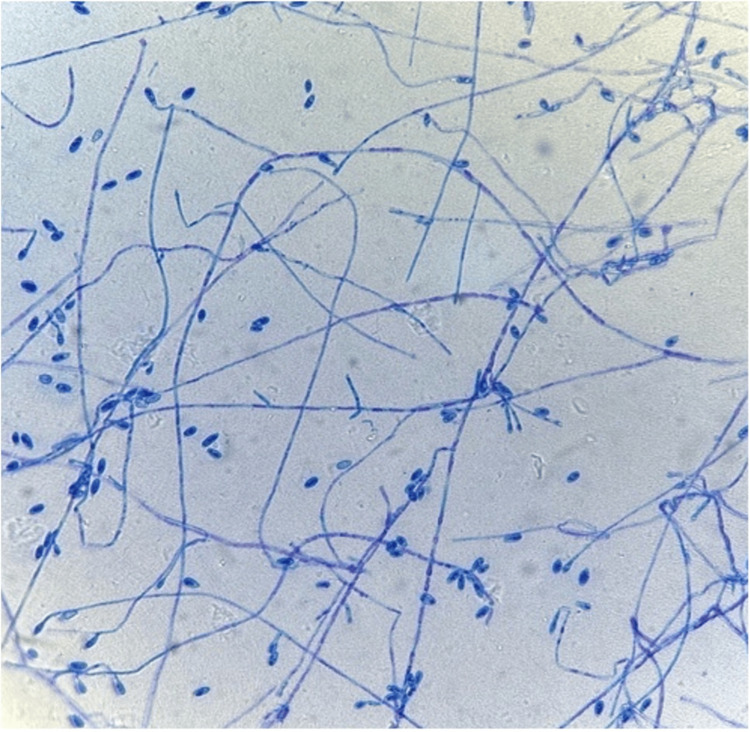
Microscopic appearance after lactophenol blue staining, demonstrating smooth, truncated conidia and slender, elongated annellides (culture on SC agar, six days of incubation at 37°C) SC: Sabouraud culture

**Figure 4:**
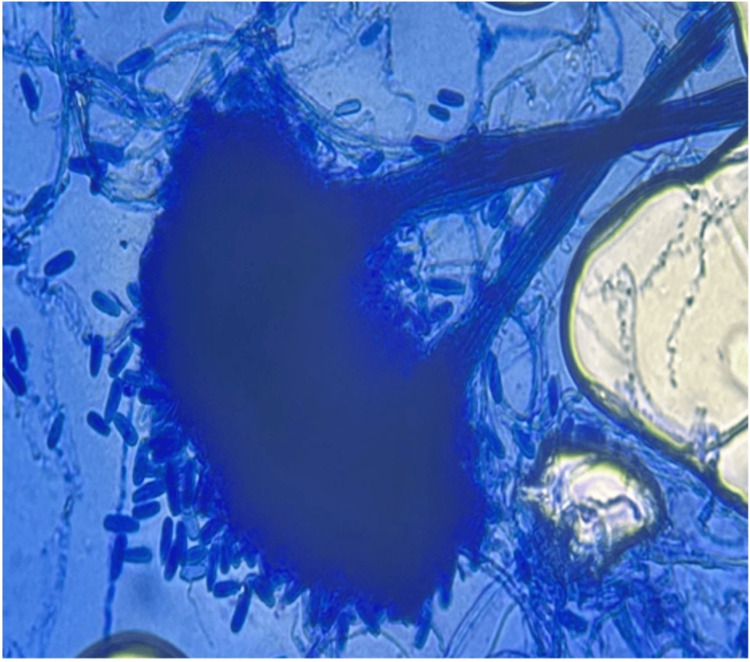
Microscopic appearance after lactophenol blue staining showing the Graphium stage, characterized by the aggregation of annellides into a coremia-like structure (synnemata) and the presence of elongated cylindrical conidia (culture on malt medium, 10 days of incubation at 37°C)

Microscopic analysis showed septate hyaline hyphae, numerous truncated oval conidia, and slender, elongated annellides (Figures 3-4).

These morphological characteristics allowed for the presumptive identification of the fungus as a member of the *Scedosporium* sp. complex. Identification of the fungus was confirmed by MALDI-TOF mass spectrometry, which assigned it to the genus *Scedosporium*.

The presence of the pathogen was also confirmed on a second sample. Further patient history revealed no recent exposure to drowning, whether in ponds or swimming pools, nor any traumatic inoculation in the preceding months. The patient was started on oral voriconazole at a dose of 200 mg twice daily.

Marked clinical and biological improvement was observed within 72 hours of treatment, including regression of clinical signs and a decrease in CRP levels. Treatment was continued for a total duration of three months. Monthly blood tests were performed to monitor treatment tolerance. A third auricular swab, obtained during a follow-up consultation, returned negative, confirming resolution of the infection.

## Discussion

Historically, *Scedosporium* spp. were part of the *Pseudallescheria*/*Scedosporium* complex, divided into two genera based on their mode of reproduction (sexual vs. asexual). Advances in molecular biology have since led to their reclassification under a single genus. The term "*Pseudallescheria*" is no longer in use [4]. The genus *Scedosporium* now includes more than 10 recognized species, such as *Scedosporium angustum*, *Scedosporium apiospermum*, *Scedosporium aurantiacum*, *Scedosporium boydii*, *Scedosporium cereisporum*, *Scedosporium dehoogii*, *Scedosporium desertorum*, *Scedosporium ellipsoideum*, *Scedosporium fusoideum*, *Scedosporium minutisporum*, *Scedosporium rarisporum*, and *Scedosporium sanyaense* [5]. Studies from several countries have shown that these species preferentially thrive in human-influenced environments such as agricultural soils, public parks, playgrounds, wastewater, and polluted lands [5]. In contrast, their presence is rarely observed in natural or forested ecosystems, suggesting a close relationship with anthropogenic environments. Among the most frequently isolated species are *Scedosporium apiospermum*, by far the most common in several regions, as well as *Scedosporium boydii*, *Scedosporium aurantiacum*, *Scedosporium dehoogii*, and *Scedosporium angustum* [5]. Infections caused by these fungi have also been reported following extreme weather events, such as tsunamis, highlighting the potential for massive accidental exposure to these environmental pathogens [6].

The primary mode of transmission of scedosporiosis is traumatic inoculation of conidia from a contaminated environmental source. Transmission generally occurs through traumatic inoculation via the skin or soft tissues following injury, or through inhalation or aspiration of contaminated water, particularly in cases of near-drowning [7]. Infection with *Scedosporium* spp. can present with a wide range of clinical manifestations, from localized cutaneous or soft tissue infections to severe disseminated disease. These manifestations have been observed in both immunocompromised and immunocompetent individuals, especially in contexts such as transplantation, trauma, pulmonary diseases, or near-drowning incidents [7]. In patients with cystic fibrosis, *Scedosporium* spp. constitute the second most frequently isolated fungal agent after *Aspergillus fumigatus* [8].

The patient's immune status and portal of fungal entry appear to play significant roles in the clinical evolution of *Scedosporium* infections. Immunocompetent patients may be asymptomatically colonized or present with localized infections such as cutaneous infections, keratitis, mycetomas, or muscular, articular, and bone infections [9]. In immunocompromised patients, *Scedosporium* spp. cause more severe, often invasive infections affecting the central nervous system (including brain abscesses and meningitis) and may progress to disseminated infection involving multiple organs. They can also cause endocarditis, particularly in patients with intracardiac devices [9].

Identification by MALDI-TOF mass spectrometry suggested that the isolate belongs to the genus *Scedosporium* with a score of 1.77. According to the manufacturer’s criteria, this score is below the threshold generally required for reliable species-level identification but remains acceptable for genus-level identification [10]. This result highlights a significant limitation of the method, primarily due to the insufficient coverage of commercial databases, which lack sufficiently comprehensive and representative reference spectra, particularly for filamentous fungi. However, enrichment of the database with well-characterized clinical isolates can significantly improve identification performance.

Regarding otomycoses, *Scedosporium* spp. have been identified as a rarely implicated etiological agent. An analysis of 14 published clinical cases demonstrates wide variability in age, immune status, clinical presentation, and outcomes. Patients’ ages ranged from four to 81 years, with a mean age of approximately 42 years. Males appeared to be more frequently affected than females. Regarding immune status, 57.1% of otomycosis cases occurred in immunocompetent individuals. Conversely, 42.9% of cases had comorbidities, predominantly diabetes mellitus. Only one case involved severe immunosuppression, combining hemophilia A, HIV infection, and hepatitis C. Clinically, the most frequent presentation in patients with *Scedosporium otomycosis* was otorrhea, observed in the majority of cases and often associated with otalgia. The most frequent complications were bone osteolysis and mastoiditis. Neurological complications were also reported, including brain abscesses, intracranial dissemination, intracranial hypertension, and facial paralysis. Perichondritis and ethmoido-maxillary sinusitis were less frequently described. Direct mycological examination was positive in 57.1% of cases. Local predisposing factors for infection were identified in 42.9% of cases, mainly involving the presence of tympanostomy tubes, cholesteatomas, or surgical history, particularly mastoidectomy.

Additionally, one case reported a bacterial co-infection with *Escherichia coli*. Surgical intervention (mastoidectomy, drainage of an extradural abscess, ventriculoperitoneal shunting, craniectomy, surgical debridement, tympanic surgery) was performed in 64.3% of cases. Simple incision and drainage of a conchal bowl abscess was carried out in two separate cases. Voriconazole was the most frequently prescribed antifungal agent, used in eight cases (57.1%), either as monotherapy or in combination with other antifungal drugs. Other antifungal agents employed included amphotericin B, miconazole, clotrimazole, ketoconazole, and econazole, as well as topical Castellani solution and resorcinol. Clinical outcomes were favorable in 71.4% of cases, whereas 21.4% of patients died, mainly in the context of severe cerebral involvement or significant comorbidities. These data highlight the invasive potential of *Scedosporium* spp., even in immunocompetent patients, and underscore the importance of early diagnosis and combined surgical and targeted antifungal therapy to optimize prognosis.

Management of infections caused by *Scedosporium* remains challenging due to its resistance to many antifungal agents. Currently, voriconazole is considered the most effective antifungal against these strains, followed by posaconazole [11]. In contrast, isavuconazole exhibits higher minimum inhibitory concentrations, limiting its clinical use [12]. To date, no randomized clinical trials have been conducted to treat scedosporiosis. Current treatment recommendations are primarily based on in vitro studies and observational cohort studies. Several studies have demonstrated that voriconazole significantly improves survival compared to amphotericin B [13]. Combination antifungal therapies have been employed in cases of treatment failure or intolerance, often involving voriconazole in conjunction with agents such as terbinafine, echinocandins, miltefosine, or even amphotericin B, with variable outcomes [14]. However, these approaches are mostly anecdotal and lack strong evidence. In cases of voriconazole intolerance or resistance, posaconazole has been used with promising results [15], although these findings require confirmation in larger prospective studies. Antifungal susceptibility testing is therefore of particular interest in infections caused by *Scedosporium* spp., as these fungi may show variable susceptibility to antifungal agents. It may help guide therapeutic choice and select the most appropriate antifungal treatment. In our case, antifungal susceptibility testing was not performed before treatment initiation, which represents a limitation of this report. However, voriconazole was chosen after the identification of *Scedosporium* spp., based on its recognized activity against this genus and the known poor susceptibility of these species to amphotericin B. Biological monitoring is useful for follow-up of NOE, particularly for assessing treatment response. Inflammatory biomarkers, such as CRP and leukocyte count, can complement clinical assessment. In our case, the normalization of inflammatory biomarkers during follow-up was consistent with the improvement of clinical symptoms, including regression of otalgia and otorrhea. Therefore, these biomarkers may serve as useful adjuncts for monitoring therapeutic response.

The clinical characteristics of reported cases of otomycosis caused by *Scedosporium* spp. identified in the literature are summarized in Table 1.

**Table 1:** Clinical cases of otomycosis caused by Scedosporium spp. reported in the literature AIDS: acquired immunodeficiency syndrome, CD4+: cluster of differentiation 4 positive, EAC: external auditory canal, VP: ventriculoperitoneal, IV: intravenous, ESBL: extended-spectrum beta-lactamase

Patient	Author, year	Age, gender	Comorbidities	Clinical features	Complication	Potential local factor	Mycological direct examination	Surgical therapy	Antifungal therapy	Outcome
											1	Yao and Messner, 2001 [16]	21, male	Hemophilia A, AIDS (CD4+ 90/mm³) and C hepatitis	Unilateral otalgia and ear bleeding for six weeks, local signs of mastoid, granulation tissue in the EAC	Osteolysis of the EAC, mastoiditis, facial nerve palsy, invasion of dura mater	No	Positive	Repeated surgical debridement: mastoidectomy, facial nerve decompression	Amphotericin B and ketoconazole then miconazole	Death
											2	Bhally et al., 2004 [17]	8, male	No	Recurrent otitis for seven years, earache, white debris in the EAC	No	No	Negative	Myringotomy	Topical clotrimazole, four weeks	Cured
3	Acharya et al., 2006 [18]	12, female	No	Recurrent unilateral otorrhea since childhood, otalgia, meningeal syndrome, intracranial hypertension	Mastoiditis cerebral dissemination: extradural abscess, multiple temporal abscesses	Cholesteatoma	Negative	Radical mastoidectomy, extradural abscess drainage	Voriconazole six months	Death
4	Baumgartner et al., 2007 [19]	62, female	No	Unilateral recurrent otorrhea since 57 years, perichondritis, conchal bowl abscess, and superficial skin necrosis	Mastoiditis	Radical mastoidectomy for treating cholesteatoma	-	Incision and drainage of conchal bowl abscess	Voriconazole six months	Cured
											5	Baumgartner et al., 2007 [19]	67, female	No	Recurrent unilateral intermittent otorrhea, acute abscess of conchal bowl and meatus with skin necrosis	Perichondritis	Radical mastoidectomy for treating cholesteatoma	Positive	Incision and drainage of conchal bowl abscess	Voriconazole six months	Healing with small postauricular fistula
6	Sai Kiran et al., 2007 [20]	21, female	No	Unilateral otalgia, otorrhea, impaired hearing for six months, intracranial hypertension, hemiparesis, cerebellar syndrome	Cerebral dissemination: cerebello-pontine angle lesion, brainstem edema, petrous apex erosion	Mastoidectomy 8 years before (chronic suppurative otitis media)	Positive	Neurosurgical treatment: VP shunt, craniectomy, lesion resection	Amphotericin B and ketoconazole	Death											
7	Vasoo et al., 2008 [21]	51, male	Diabetes	Unilateral otalgia and greenish otorrhea for a month, granulation in EAC	Osteolysis of temporal bone, mandibular condyle and pterygoid process, soft tissue involvement (parotid, muscle), facial nerve palsy	No	Negative	No	Voriconazole	Cured											
8	Buzina et al., 2008 [22]	65, female	Diabetes	Unilateral otalgia and purulent otorrhea for years, pruritus, debris in EAC	No	Cholesteatoma	Negative	Tympanosurgery	Voriconazole	Cured
9	Bienvenu et al., 2009 [23]	72, male	Diabetes	Unilateral purulent otorrhea for six months, EAC stenosis	Osteolysis of petrous bone, ethmoido-maxillary sinusitis	No	Negative	Surgical excision of bone fragment in EAC	Voriconazole for four weeks	Cured
10	Neji et al., 2013 [24]	32, male	No	Hypoacusia for seven years, purulent otorrhea right side for four years, then left	Inflammatory EAC	No	Positive	No	Topical econazole nitrate (10 weeks)	Cured
11	Koay et al., 2015 [25]	4, male	No	Chronic otorrhea and otitis media	No	Placement of bilateral tympanostomy tubes	Positive	The retained tube was replaced with a Duravent tympanostomy tube	Clotrimazole eardrops were prescribed three times a day for 14 days	Cured
12	Wen et al., 2016 [26]	13, female	No	Severe otalgia requiring regular aspiration of the right external auditory canal	No	No	Positive	Surgical debridement	Topical Castellani and resorcinol	Cured
											13	Fuster-Escrivá et al., 2019 [27]	77, male	Diabetes, circulatory insufficiency	Unilateral otalgia, otorrhea, and left-sided facial paralysis	Otomastoiditis	*Escherichia coli *producing ESBL ear infection	Positive	No	Voriconazole IV (200 mg/12 hours) was discontinued and switched to anidulafungin because of side effects	Death
											14	Hussain et al., 2023 [28]	81, male	Diabetes	Left-sided otalgia, otorrhea, hearing loss evolving over two months, and facial weakness	Skull base osteomyelitis	No	Positive	Removal of necrotic bone and remnants of the polyp	Tigecycline and ciprofloxacin ear drops were added to the voriconazole treatment	Cured

## Conclusions

This case highlights the importance of considering a fungal etiology in patients presenting with persistent otalgia and otorrhea, particularly when symptoms do not improve despite appropriate antibacterial therapy. Although NOE is most commonly associated with bacterial pathogens, *Scedosporium* sp. should be considered in refractory or complicated cases, especially in patients with underlying risk factors such as diabetes. Early mycological examination is essential for establishing the diagnosis and avoiding delays in initiating appropriate therapy. This is particularly important because *Scedosporium* spp. may show poor susceptibility or intrinsic resistance to several antifungal agents, especially amphotericin B. Therefore, accurate fungal identification is crucial for selecting an appropriate antifungal therapy, with voriconazole remaining the preferred treatment option in many reported cases. This observation also emphasizes the need for close clinical follow-up, as fungal NOE may lead to severe local or invasive complications. A multidisciplinary approach involving clinicians, microbiologists, and radiologists is therefore necessary to improve diagnosis, treatment, and patient outcomes.
